# A Reusable Multiplayer Game for Promoting Active School Transport: Development Study

**DOI:** 10.2196/31638

**Published:** 2022-03-14

**Authors:** Teemu H Laine, Nhi Duong, Helena Lindvall, Solomon Sunday Oyelere, Stina Rutberg, Anna-Karin Lindqvist

**Affiliations:** 1 Department of Digital Media Ajou University Suwon Republic of Korea; 2 Haaga-Helia University of Applied Sciences Helsinki Finland; 3 Luleå Municipality Luleå Sweden; 4 Department of Computer Science, Electrical and Space Engineering Luleå University of Technology Skellefteå Sweden; 5 Department of Health, Education and Technology Luleå University of Technology Luleå Sweden

**Keywords:** gamification, active school transport, physical activity, formative evaluation, architecture, mobile phone, web browser

## Abstract

**Background:**

Most children and adolescents in Sweden do not meet the recommended daily physical activity levels of the World Health Organization. Active school transport (AST) and gamification are potential methods for increasing children’s daily physical activity. We previously developed a game named Tic-Tac-Training for promoting active transport at workplaces; however, the game has not been applied to AST.

**Objective:**

The objectives of this study are to investigate how Tic-Tac-Training functions to promote AST among schoolchildren in northern Sweden, improve the game to be more suitable for schoolchildren, and construct a road map for future development based on children’s ideas.

**Methods:**

First, we developed Tic-Tac-Training using the Scrum agile software development method. Second, we conducted a questionnaire-based formative evaluation of the game with schoolchildren (n=16; 9/16, 56% male; 6/16, 38% female; and 1/16, 6% other aged 11-12 years) in Luleå, Sweden. Third, we conducted focus group interviews with 33 children (13/33, 39% male and 20/33, 61% female aged 12-13 years) to gather ideas for gamifying AST. We mapped the interview results to the Octalysis gamification framework and established a road map for future development.

**Results:**

The formative evaluation revealed several issues, including a lack of interesting game features, lack of support for continuous engagement, disliked competitive features, and lack of incentives for discourse and participation. New features such as rewards, collectibles, and levels were implemented based on the results. The focus group interviews revealed additional ideas for gamifying AST, such as using avatars, in-game currency and trading, and context-sensitive tasks.

**Conclusions:**

The results have several potential impacts on how reusable, gamified AST interventions can be developed and what kind of gamification elements schoolchildren in northern Sweden wish to see. These results can interest game researchers and teachers who wish to apply gamification in school contexts. Finally, we aim to continue developing the game based on the road map.

## Introduction

### Physical Activity and Active School Transport

Considerable health benefits are associated with increased physical activity, such as cardiorespiratory and muscular fitness and a positive effect on weight status [1-3]. Furthermore, there is evidence concerning the positive impact of physical activity on cognitive abilities and prosocial behavior [4]. However, most children and adolescents worldwide do not reach the recommendation of daily physical activity from the World Health Organization [1,5].

A solution is to use active transportation daily, which has substantial positive impacts [6]. Moreover, physically active children and adolescents are more likely to adopt healthy behaviors, and a physically active lifestyle in childhood often transfers to adulthood [7-9]. Overall, adequate physical activity in childhood can lead to healthier lives in adulthood through avoidance of adverse health effects related to obesity and through continuity of regular physical activity behavior [10]. The health benefits of walking and cycling for transport entail a significant reduction in risks for cancer mortality, cardiovascular disease, and obesity morbidity [11,12]. Although active transport involves risks such as traumatic accidents and exposure to poor air quality [13,14], the health benefits of cycling are significantly larger than the risks of cycling when compared with car driving [3,15,16]. There is also climate protection and environmental benefits accrued to active transport–based physical activities [17] and the possibility of decreasing traffic around schools [18].

Despite the numerous benefits of active transport to increase physical activity, only 57% of children aged 6-15 years in Sweden use active transport to school during spring and fall, with an even lower frequency of 48% during winter [19]. The trend of decreasing the use of active transport among children and adolescents is global, and it is expected to continue if interventions are not made to change this trend [20-22]. Increasing daily routines for physical activity, such as using active school transport (AST), is also a prioritized area in the global plan of the World Health Organization for increasing physical activity [23].

This study focuses on AST, in which nonmotorized methods are applied to transportation between homes and schools. Thus, AST can increase much-needed daily physical activity among children. However, adopting AST can be challenging given that motorized transportation has many attraction points, such as convenience, weather independence, and avoiding trip chaining [24]. In contrast, AST requires physical exertion and is affected by weather conditions, the latter of which can be a significant factor in northern Sweden. Moreover, parents’ concerns, such as the availability of infrastructure, distance, time, traffic safety, and compatibility with other activities requiring transportation, form additional barriers that AST solutions must overcome [25,26]. Despite these challenges, successful AST in winter is possible as long as the motivational aspects of AST (eg, promoting togetherness and involvement) are adequately realized [27].

### Gamification of Physical Activity and AST

Gamification refers to applying elements of games, such as rules of playing, badges, points, and leaderboards, in nongame contexts. Games have been shown to possess strong motivators that drive a person’s desire to engage in unappealing activities [28] such as cycling during snowy winter conditions [27] and using active transport instead of motorized transport [29]. Moreover, several notable models and frameworks such as Octalysis [30], Mechanics-Dynamics-Aesthetics [31], and Hexad [32] have been proposed to assist researchers and practitioners in applying gamification to diverse areas. In addition, widely spread commercial games such as Pokémon GO have been shown to positively influence the physical activity and health of both child and adult players [33-35].

Recent studies have suggested using gamification to promote physical activity among children and adolescents to encourage and motivate behavior change toward a physically active lifestyle. Quintas-Hijos et al [36] explored the use of gamification in the context of physical education in fifth and sixth grade. The results affirmed that gamification provided a greater overall positive feeling, fun, creative inspiration, autonomous learning, motivation, and digital leisure alternatives. Corepal et al [37] carried out a gamified intervention at a secondary school in Northern Ireland. The StepSmart Challenge was an intervention for adolescents to change their physical activity behavior through team and individual competition using motivation and incentives. Another study used weekly challenges among students where they earned badges, trophies, and rewards after the intervention to improve cardiorespiratory fitness and reinforce positive physical activity behavior [38]. In another study, Lindberg et al [39] developed and evaluated a sensor-driven board game in which players competed against each other by solving physical and pedagogical missions to complete moves on a large game board. The results indicated increased learning efficiency, player engagement, and exertion. Finally, Comeras-Chueca et al [40] conducted a systematic review of the use of active video games for overweight and obese children and adolescents. Their findings showed that active video games have clear positive effects on BMI and body fat percentage, but their effects on muscular fitness, fat-free mass, waist circumference, and motor competence remain unclear.

In addition to supporting children’s physical activity, gamification has been applied to promote AST with notable benefits [41-44]. Coombes and Jones [41] found evidence that children who engaged in gamification that encouraged them to walk and cycle to school using tracking technology with a reward scheme increased their AST time and physical activity level. Specifically, there was an increase in weekly active travel and a positive association between moderate to vigorous physical activity during school commuting times and the number of days children used the gamified system. In another study built on gamification elements, the participants recorded their walking and cycling travels by tapping “Beat Boxes” (radio-frequency identification scanners installed at various locations in the game area) and received points and incentives [43]. The results of this study showed increased physical activity levels among the participants. Another study on the same project showed that collective rewards, social influence, and exploration were key motivating factors for engagement with the intervention [44].

In 2020, we presented a novel distributed multiplayer game, Tic-Tac-Training, for promoting active transport at workplaces [45]. Tic-Tac-Training is an adaptation of the Tic-Tac-Toe game in which players compete against other teams by completing various tasks related to active transportation and sustainable working habits. The game can be customized by defining the tasks that the players are to complete. This way, the game system could be applied to a wide range of contexts, such as AST for children.

### Context of the Study

This study focuses on northern Sweden, particularly the municipality of Luleå, which has a population of 77,832, of which 7492 were pupils attending grades 1-9 in the academic year 2019-2020 [46]. The municipality has a wide network of bicycle and pedestrian roads and strong expertise in maintaining the roads in winter conditions (eg, plowing and spreading sand and salt), making motorized and nonmotorized transportation feasible throughout the year.

Despite the feasibility of nonmotorized transportation, the number of children using AST has been low. The situation has recently improved owing to efforts to promote AST in schools. The fifth (SR) and sixth (AKL) authors implemented a previous intervention to promote AST based on empowerment and nondigital gamification [47]. To scale the intervention, a digital gamified AST tool would be vital.

This study is part of the multidisciplinary project Sustainable Innovations for Children Transporting Actively. One of the project’s aims is to create new knowledge about applying games to engage children in behavior change in favor of AST. In addition, the project co-designs curriculum-based learning activities with teachers, pupils, and parents to motivate and engage AST.

### Aim and Contributions

We aim to investigate how the Tic-Tac-Training game is suitable for promoting AST among schoolchildren, how its suitability can be improved, and what gamification elements schoolchildren would like to have in the AST game. Consequently, we make the following contributions: (1) a description of the game and its technical implementation, (2) a formative evaluation with schoolchildren in Luleå to test the suitability of the game concept for AST, (3) a refinement of the game by adding features based on the formative evaluation results, (4) a presentation of other AST gamification ideas gathered from schoolchildren and mapping of the ideas to the Octalysis gamification framework [30], and (5) a road map for the next iteration of development of Tic-Tac-Training based on these ideas.

## Methods

### Research Design

**Figure 1:**
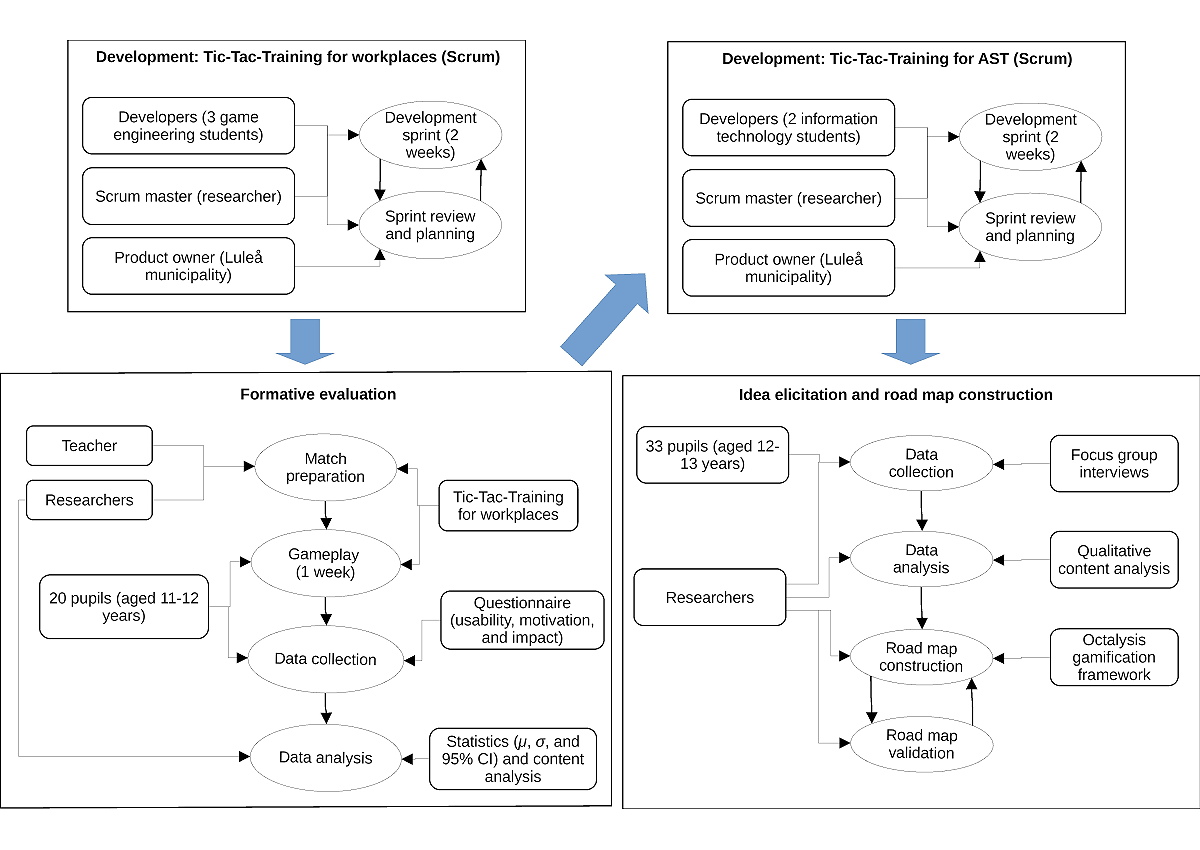
Research design. AST: active school transport.

This study followed a mixed methods approach, comprising both quantitative and qualitative methods. The core part of the study is an iterative, user-centered development of the Tic-Tac-Training game building on the contributions of end users, university students, and elementary school pupils. In addition, we conducted a formative evaluation of the game using a questionnaire, as well as focus group interviews to explore the pupils’ experiences and ideas for the gamification of AST. The overall research design of this study is shown in Figure 1. The 4 stages of research are explained in detail in the following sections followed by an ethics statement.

### Game Development: Tic-Tac-Training for Workplaces

Tic-Tac-Training was originally developed for workplaces with a user-centered design method whereby the designers iteratively constructed a series of design prototypes in the context of the Luleå municipality [45]. The workplace version of Tic-Tac-Training was implemented using the Scrum agile development method [48] whereby biweekly scrum sprint review and planning meetings were arranged between the developers (3 university students in game engineering), the scrum master (the first author), and the product owner (Luleå municipality and the third author).

### Formative Evaluation

The purpose of the formative evaluation [49] was to measure the perceptions of a group of elementary school pupils in Luleå of the Tic-Tac-Training game, which would guide the iteration of the game development process. In particular, we sought to understand how children react to a game created to promote active transport at workplaces by exploring the aspects of usability, motivation, and expected impact.

#### Participants

In total, 20 voluntary participants were recruited among sixth-grade pupils by a teacher at a Luleå-based school in February 2020. The number of participants was deemed to be sufficient for a formative evaluation to identify most shortcomings and problems; in the classic usability testing guideline, 5 participants are recommended as an adequate number, and 15 is found to be an optimal number of participants for a user test in a medium to large project [50]. The ages of the participants were between 11 and 12 years, with the gender distribution of the questionnaire respondents being 56% (9/16) male, 38% (6/16) female, and 6% (1/16) who chose *other* as their gender. As the participants were minors, we obtained permission from their parents.

#### Data Collection Instrument

We developed a questionnaire (Multimedia Appendix 1) comprising demographic information (school, age, and gender) and three sections comprising 5-point Likert scale questions: usability, motivation, and impact. In addition, open fields were provided to the participants to motivate their answers and for them to provide comments freely. The usability section measured the overall usability of the game using questions that were adopted from the System Usability Scale [51] and Net Promoter Score [52]. The motivation section gauged the enjoyment factors, if any, in the gameplay. The impact section measured the impacts of the game on areas such as physical movement, environment, and fellowship in the class. The questionnaire was developed in Swedish, and we asked a teacher to validate the language before the questionnaire was administered. Finally, the questionnaire did not contain any personally identifiable information about the participants.

#### Match Preparation

Researchers and a teacher created a total of 24 tasks in Swedish. The tasks covered various activities such as cycling, walking, playing outdoors, and convincing adults at school to perform AST. Instructional videos were created for the teacher to learn about the game and its features, including a tutorial on creating a match. The teacher created an account for herself in January 2020 and was promoted to the match creator role by the game administrator. The teacher then created a match for her pupils using the previously created tasks.

#### Data Collection and Analysis

The gameplay and data collection took place in February 2020. We first obtained permission from the participants’ parents for their children to participate in the formative evaluation. The teacher then provided the pupils with instructions to create a player account, after which she configured and started a 1-week–long match for the pupils with 5 teams of 4 pupils each. The teacher explained how the gameplay worked and answered the pupils’ questions. The teacher was asked to contact the researchers if any technical problems or questions arose that she was not able to answer. After the gameplay ended, the pupils were asked to fill in the questionnaire delivered through Google Forms. In total, 16 pupils replied to the questionnaire. The questionnaire data were analyzed using descriptive statistics (mean, SD, and CIs) of the Likert data and simple content analysis of the open-field data.

### Game Development Method: Tic-Tac-Training for AST

On the basis of the formative evaluation findings, 2 university students in information technology continued the development by adding new features that would make the game more suitable for AST. The same Scrum method [48] was used as described above. The 2 students were different from the original group of student developers.

### Idea Elicitation and Road Map Construction

In 2017, 2 classes with 40 children aged 12-13 years participated in the nondigital AST intervention by the fifth and sixth authors. After the intervention, 33% (13/40) male and 50% (20/40) female children gave their informed consent to participate in focus group interviews with 4-7 students in each group. They were asked to discuss their experiences with participating in the intervention concerning photos they had taken during the intervention period; the results were published elsewhere [27]. In addition, we asked the participants to discuss their ideas and thoughts about developing a digital game to support the AST intervention; these data are presented in this study.

The focus group interview data were transcribed and analyzed using qualitative content analysis [53] by the fifth and sixth authors to identify gamification ideas. Once the ideas were recorded, the first author analyzed them through the lens of the Octalysis gamification framework [30]. This framework is well-known in the field of gamification and comprises 8 *core drives* into which various gamification elements can be placed. The first author mapped the children’s ideas to the core drives and then, based on the ideas, derived concrete feature suggestions for each core drive that could be implemented in the next iteration of development. The other authors validated the mappings and feature suggestions, resulting in the final feature road map.

### Ethics Statement

This study was conducted in accordance with the ethical principles within Swedish law for research and the Declaration of Helsinki of the World Medical Association. This study was approved by the Regional Ethical Board of Umeå, Sweden (2018-10-31M). The participating students and their parents provided informed consent. All students were informed about the possibility to participate or not, as well as that they could decide to withdraw their participation at any time.

## Results

### Tic-Tac-Training for Workplaces

In this section, we describe the details of the implementation of the workplace version of Tic-Tac-Training before presenting the formative evaluation results. First, we describe the game concept followed by the game’s architecture and implementation details.

#### Game Concept

##### Basic Gameplay

**Figure 2:**
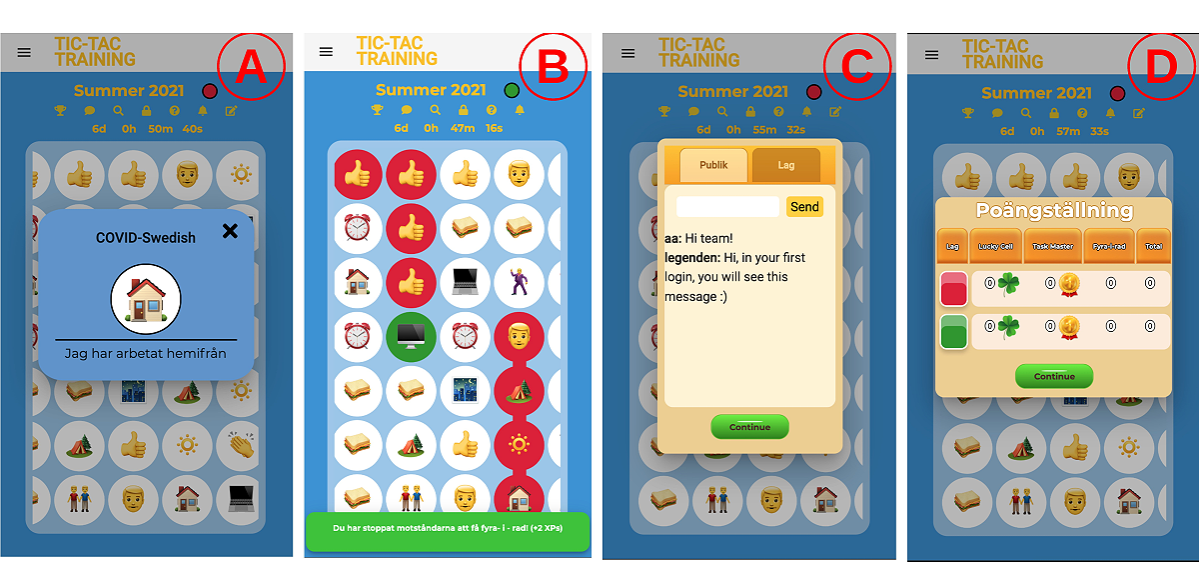
Tic-Tac-Training gameplay screens. (A) Task details, (B) game board where the green team blocked the red team, (C) in-game chat, (D) match statistics.

The purpose behind developing Tic-Tac-Training was to motivate people to become physically more active. The game was designed to offer competitive and collaborative gameplay by expanding upon the basic concept of the Tic-Tac-Toe board game. Unlike Tic-Tac-Toe, players play in teams without turns and earn points by completing various tasks assigned to the game board cells. Before completing the task, players can open the description box of the task to understand the requirements (Figure 2A). Completing 4 cells in a diagonal, horizontal, or vertical row yields the team a point. The players can also block other teams. These cases are illustrated in Figure 2B. A match ends when the time runs out or when one of the ending conditions is met (see *Match and Task Creation*).

**Figure 3:**
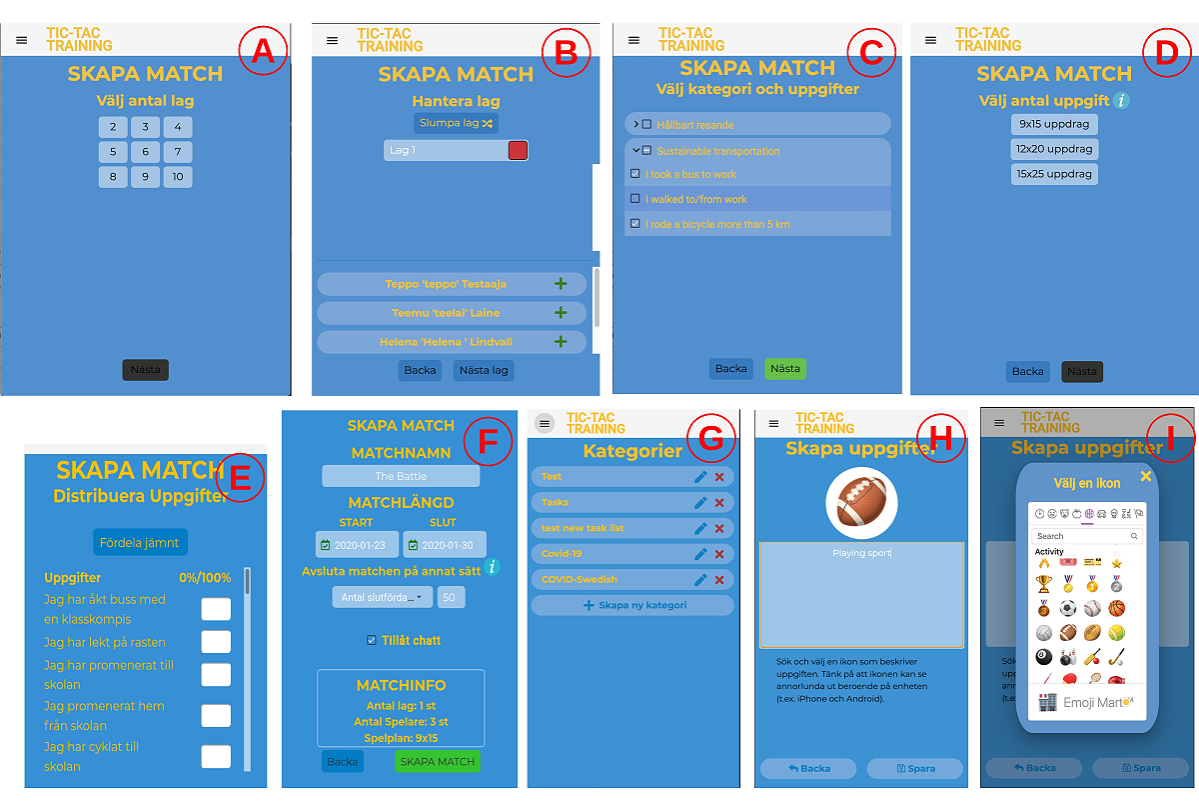
Tic-Tac-Training match and task creation screens. (A) Selecting how many teams will play; (B) assigning players to teams; (C) selecting tasks for the match; (D) selecting the game board size; (E) selecting distribution of the tasks; (F) choosing match name, period, ending condition, and chat; (G) managing task categories; (H) creating a new task; (I) choosing an icon for a new task.

The player profile contains basic information about the players, such as their name, nickname, email address, picture, and a short description. When the player selects a match after log-in, it is opened with a match timer and several tools such as (1) a chat room where the player can communicate with their team as well as with other teams (Figure 2C), (2) a leaderboard where players can see their team’s position relative to other teams (Figure 2D), and (3) a task filter that allows the player to highlight only the desired tasks on the game board.

##### Match and Task Creation

A match can be created and modified by a match creator (eg, a teacher). The basic match settings are the number of teams (Figure 3A); team members, colors, and names (Figure 3B); tasks for the match (Figure 3C); game board size (Figure 3D); tasks and their distribution on the game board (Figure 3E); and match name, duration, end condition, and whether to enable chat (Figure 3F). A match can be set to expire after some time when the number of completed rows reaches the set target or when the number of completed cells reaches the set target. Finally, the match creator can modify some match settings (eg, team compositions and match-ending conditions).

The match creator creates match tasks by first selecting a suitable task icon from a searchable list of emojis (Figure 3I) and then adding a description for the task (Figure 3H), such as *Ride bicycle to school*, *Take a walk during lunch break*, or *Run two kilometers*. Moreover, the created tasks can be grouped into categories (Figure 3G), enabling the selection of several tasks at once during match creation. Finally, all the created categories and tasks can be reused in matches and optionally shared with other match creators.

#### Architecture and Implementation

**Figure 4:**
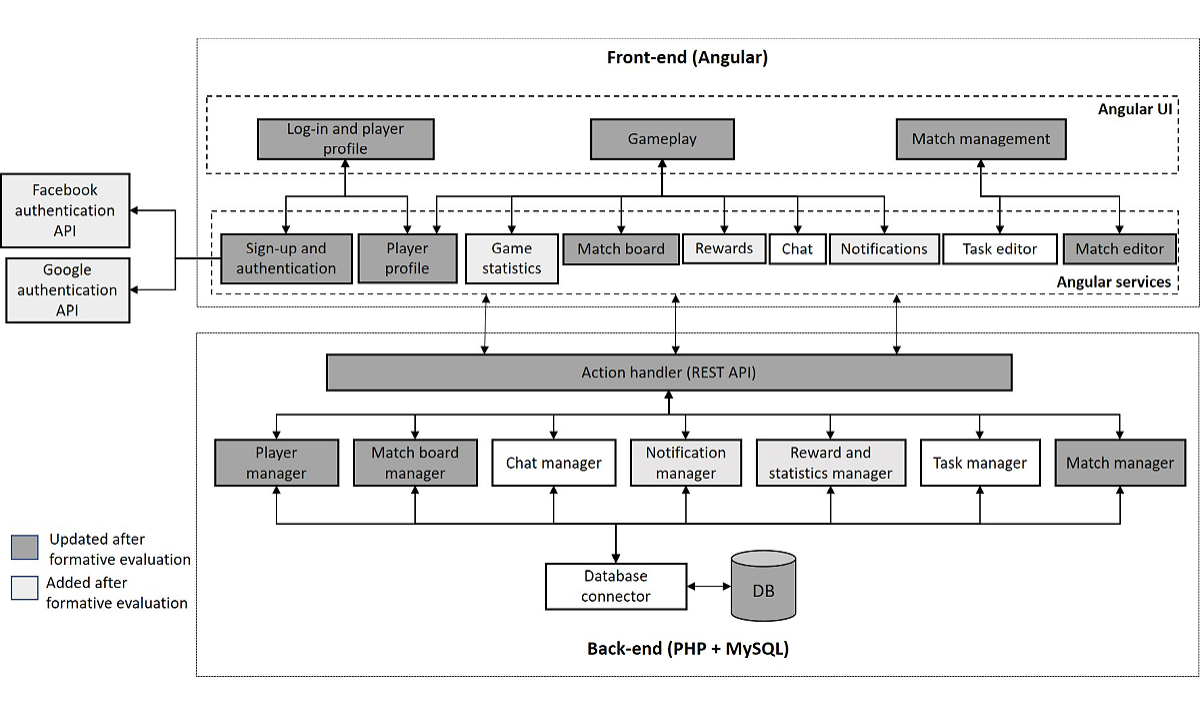
Tic-Tac-Training architecture based on Angular, PHP Hypertext Preprocessor, and MySQL. API: application programming interface; DB: database; PHP: hypertext preprocessor; REST: Representational State Transfer; UI: user interface.

Tic-Tac-Training was developed on a client–server architecture where the web-based client can be accessed with a web browser. Figure 4 depicts the game architecture we implemented using Angular, PHP Hypertext Preprocessor, and MySQL technologies. Firebase (via the AngularFire library, Google Inc) was used to store chat messages and notifications, and Facebook and Google application programming interfaces (APIs) were used for optional log-in services. We divided the game features into Angular services accessed by the user interface (UI) components in the front-end architecture. For example, the UI components related to gameplay use Match Board (for overall gameplay management), Rewards (for Lucky Cells, Task Masters, and overall game statistics), Game Statistics, Chat, Notifications, and Player Profile (for accessing player information during a match). The Login and Player Profile UI allows players to register and log-in as well as view and edit their profiles. The Angular services communicate with the back-end managers over a Representational State Transfer API. The back-end Action Handler works as a front controller that delegates requests to appropriate managers that perform various database operations on the game data.

The UI is responsive to web browsers on desktop computers, tablets, and smartphones. We implemented the UI using HTML templates that we dynamically updated from the Angular components. In addition, we implemented a notification system based on the Web Notification API.

### Formative Evaluation

This section reports 16 schoolchildren’s perceptions of Tic-Tac-Training for workplaces. The results of our analysis are presented according to the questionnaire sections: usability, motivation, and impact.

#### Usability

The items on the game’s usability, presented in Table 1, were mostly agreed with. For example, most participants agreed that the game was easy to use (1; *μ*=3.87; σ=1.03). In addition, they agreed that it was easy to understand how the game worked (2; μ=3.56; σ=1.26). Further support for adequate usability was evident in the following statements that most participants disagreed with: the game was too complicated (3; μ=1.75; σ=0.86), the game was difficult to use (6; μ=1.94; σ=1.34), they would need someone’s help to use the game (7; μ=1.75; σ=1.00), the game was hard to learn to play (9; μ=1.69; σ=1.08), and the game was too troublesome (11; μ=2.37; σ=1.50). Given that the usability results were generally positive, it was surprising to us that the participants somewhat disagreed that they would like to play the game again (10; μ=2.56; σ=1.50). Aligned with this, the statement of the Net Promoter Score—*I can recommend the game to others* (4)—was also somewhat disagreed with (μ=2.37; σ=1.36).

**Table 1:** Responses to the usability statements (derived from the System Usability Scale [51] and Net Promoter Score [52]).

Item number	Statement	μ (95% CI)	σ
1	The game was easy to use.	3.87 (3.32-4.42)	1.03
2	It was easy to understand how the game worked.	3.56 (2.89-4.23)	1.26
3	The game was too complicated.	1.75 (1.29-2.21)	0.86
4	I can recommend the game to others.	2.37 (1.65-3.09)	1.36
5	I am satisfied with the game as a whole.	3.31 (2.77-3.85)	1.01
6	The game was difficult to use.	1.94 (1.23-2.65)	1.34
7	I think I need someone’s help to use the game.	1.75 (1.22-2.28)	1.00
8	I think most people would learn to use the game quickly.	3.69 (3.08-4.3)	1.14
9	It was hard to learn to play the game.	1.69 (1.11-2.27)	1.08
10	I would like to play the game again.	2.56 (1.76-3.36)	1.50
11	The game was too troublesome to use.	2.37 (1.57-3.17)	1.50

#### Motivation

The motivational items that promoted active transport and collaboration with classmates received the most agreement from the participants, as shown in Table 2. Specifically, the participants agreed that they wanted to play the game because it was good for the environment to walk and cycle (18; μ=4.06; σ=0.93), it was good for the body to move (17; μ=3.44; σ=1.37), and they could collaborate with classmates (13; μ=3.44; σ=1.20). In addition, the game tasks were found to be motivating to most participants (14; μ=3.06; σ=1.18). There were significant disagreements among the participants on the motivations regarding competing against others (12; μ=1.94; σ=1.12) and having the opportunity to talk, discuss, and make plans to play as a team (15; μ=2.44; σ=1.41). The fact that the game was part of the participants’ schoolwork was motivating only to a slight majority of the participants (19; μ=3.31; σ=1.35).

**Table 2:** Responses to the following motivation statements: “I enjoyed playing the game because....”

Item number	Statement	μ (95% CI)	σ
12	...we could compete.	1.94 (1.34-2.54)	1.12
13	...of the collaboration with classmates.	3.44 (2.8-4.08)	1.20
14	...of the tasks in the match.	3.06 (2.43-3.69)	1.18
15	...of the opportunity to talk, discuss, and make plans for how we would play.	2.44 (1.69-3.19)	1.41
16	...of notifications in the game (eg, when some team got four-in-a-row).	2.69 (1.92-3.46)	1.45
17	...it is good for the body to move.	3.44 (2.71-4.17)	1.37
18	...it is good for the environment to walk and cycle.	4.06 (3.56-4.56)	0.93
19	...this was part of school work.	3.31 (2.59-4.03)	1.35

#### Impact

Finally, the formative evaluation analyzed the participants’ responses regarding the perceived impacts of playing the multiplayer Tic-Tac-Training game. The results are presented in Table 3. The participants disagreed that the game contributed to increasing fellowship in the class (20; μ=2.26; σ=1.34). In addition, they disagreed that the game contributed to walking and cycling more (23; μ=2.56; σ=1.37), moving more (21; μ=2.38; σ=1.46), and improving the environment (22; μ=2.81; σ=1.38).

**Table 3:** Responses to the following impact statements: “The game has contributed to....”

Item number	Statement	μ (95% CI)	σ
20	...increased fellowship in the class.	2.25 (1.54-2.96)	1.34
21	...my increased movement.	2.38 (1.6-3.16)	1.46
22	...improvement of the environment.	2.81 (2.07-3.55)	1.38
23	...my increased walking and cycling.	2.56 (1.83-3.29)	1.37

#### Analysis of the Formative Evaluation Results

The results of the formative evaluation revealed several points for improvement in Tic-Tac-Training when repurposing it for schoolchildren. First, although usability was generally deemed to be adequate, most of the participants did not want to play the game again or recommend it to others. Some hints as to why this result was achieved can be seen in the following excerpts that were captured from the open answers of the participants when we asked them to motivate their responses to the usability questions:

It was just buggy sometimes.

Male, 11

It was just boring.

Female, 11

Boring game.

Male, 11

Bad game.

Female, 11

The game had some technical and usability glitches discovered during the test that may have triggered discontent among the participants. The perceptions of the game being boring and bad can be attributed to the fact that the game was originally developed for another kind of audience. The game was toned down in comparison with games that children typically play, which often include colorful visuals and diversity in terms of game mechanics, such as rewards, collectibles, level-ups, and badges. Another factor that could have influenced the participants’ opinion of not wanting to play the game again and their unwillingness to recommend the game to others is that they played only 1 match, which may not have been enough to form a deeper understanding of and connection with the game. Moreover, the game provided little support for maintaining interest between matches; each match was considered a new incarnation of the match board and the teams, with nothing for the player to build upon when playing multiple matches. If the game better supports the player’s profile development through points, levels, collectibles, and other mechanics, it might motivate the player to play again.

One of the core game mechanics of Tic-Tac-Training is competition between teams—competition was one of the aspects that adult players at a workplace wished to have in the game [45]. However, the participants received this game mechanic poorly; they were more motivated by collaboration with their classmates. Thus, we can conclude that the game should be playable in a noncompetitive, collaborative manner by schoolchildren. This is aligned with the results of a study on Pokémon GO suggesting that collaboration conquers competition among children [54].

The participants were not motivated by the notifications produced by the game of important events. A reason for this could be that some players missed the incoming notifications. This could have happened because (1) after a notification was dismissed, it was difficult to find it again; (2) notifications were only shown on devices that supported them (eg, Safari on Apple devices did not work with the notification system at the time of evaluation); (3) the game sent notifications seldom, such as when a team completed 4-in-a-row; and (4) if the player selected to decline notifications when the game was opened for the first time, all notifications were blocked. Moreover, the players had no way to choose which notifications to show or hide. This lack of control could have affected their overall motivation toward the feature.

Although the participants enjoyed collaborating with their classmates, they disagreed about the opportunity to talk, discuss, and make plans for playing. This is aligned with our observation that the game chat was not used much by the teams. We suspected that the players, being from the same class, had existing communication channels that they used.

Table 4 summarizes the shortcomings that our analysis revealed, along with the proposed updates to make the game more appropriate for AST. These updates were implemented in Tic-Tac-Training from April 2020 to August 2020, as detailed in the next section.

**Table 4:** Primary shortcomings identified in the formative evaluation and proposed updates.

Shortcomings	Proposed updates
Boring game	Increase the diversity of game mechanics through rewards, collectibles, and levels; update the user interface to be more appealing
Lack of support for continuous engagement	Introduce level-ups and badges that are earned only by actively participating in matches; show diverse statistics that are updated based on played matches
Disliked competition and lack of fellowship	Implement a game mode that is only based on collaboration without competition between teams
Demotivating or unnoticed notifications	Update the notification system by (1) adding more different types of notifications of various game events, (2) allowing the player to choose which notifications to receive, and (3) sending the most important notifications by email
Lack of incentives for discourse and participation	Inform the players about the existence and purpose of the chat; show statistics that motivate players to contribute to their team; allow players to use social media accounts to log-in, thereby connecting the game to their existing social media profiles

### Tic-Tac-Training for AST

**Figure 5:**
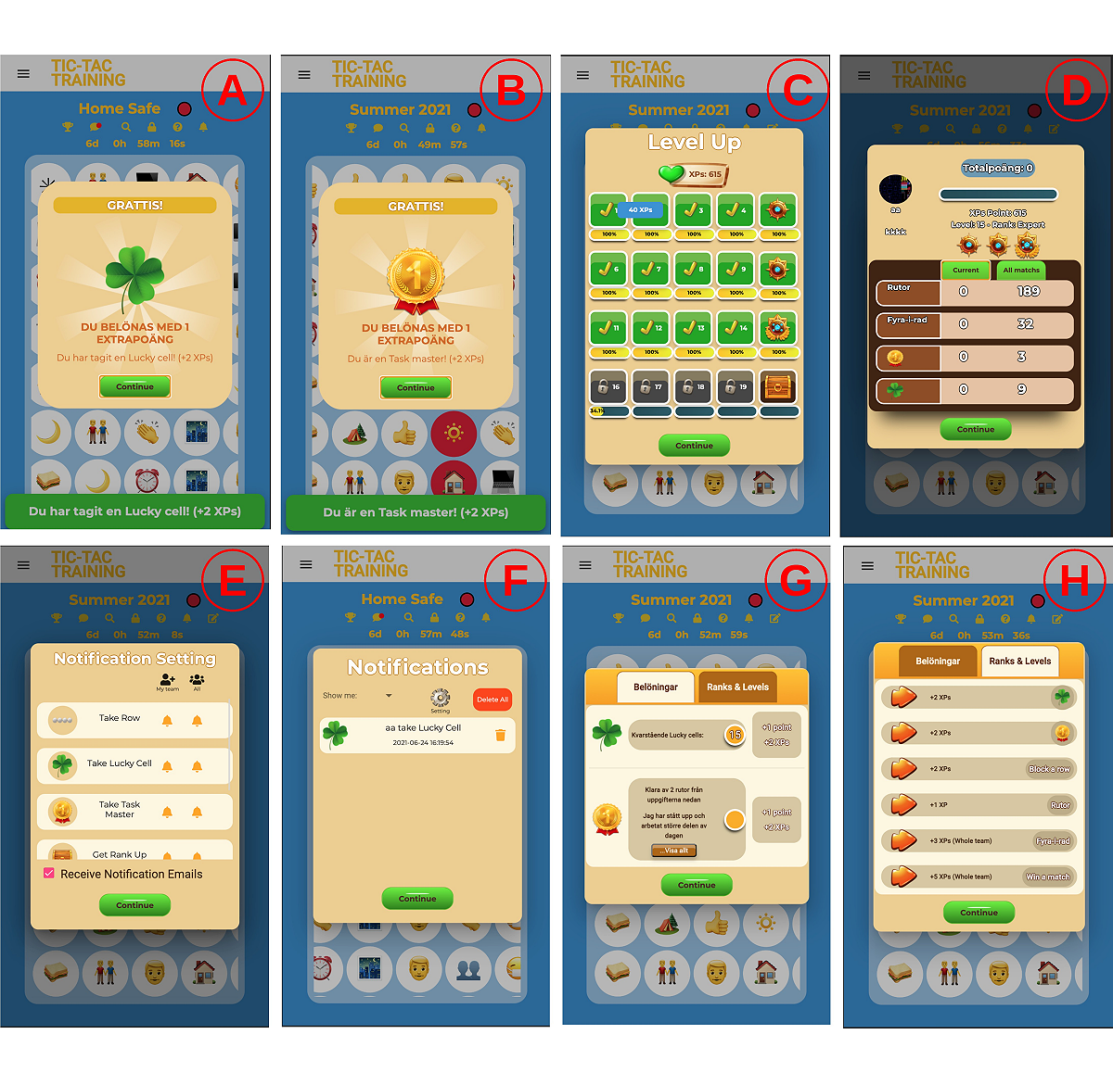
Tic-Tac-Training new features: gameplay. (A) Lucky Cell reward, (B) Task Master reward, (C) level progression view, (D) player statistics, (E) notification settings, (F) notification history, (G) information about rewards in a match, (H) information about how experience points can be earned in a match.

We developed new features to overcome the identified shortcomings, improve the overall player experience, and promote teamwork. We developed scoring, level, ranking, and reward systems to keep players motivated and engaged in long-term gameplay. We designed a reward system with two reward types: Lucky Cell and Task Master. The Lucky Cell reward is earned by capturing cells with the tag *lucky cell* (Figure 5A), which are randomly distributed on the game board. The Task Master reward is given to the first player who completes a certain number of tasks (Figure 5B). The match creator defines the type of tasks for the Task Master. All the available rewards are listed in the *Information* box of the match (Figure 5G), and the player is notified immediately after they win a reward.

We also implemented a scoring, level, and ranking system with 20 levels and 5 ranks (badges; Figure 5C). Players are awarded a new rank after every 5 levels. Players gain levels and ranks by collecting experience points (XPs) from various game activities (Figure 5H), either as individuals (eg, Lucky Cells, Task Masters, and capturing cells) or as a team (eg, 4-in-a-row and winning a match). The player’s profile presents the current rank, level, and XPs (Figure 5D).

We redesigned the UI to be more colorful, consistent, and appealing to the children (Figure 5). We used consistent colors, dialog boxes, notifications, buttons, and icons to reduce the learning curve. Moreover, we used animations in notifications and pop-up windows to draw the user’s attention and make the game look more dynamic. In addition, whenever the player completes a row, blocks a row, obtains a reward (Figure 5A and B), obtains a level-up, or captures a cell, the game responds with a short pop-up message showing the earned XPs. The XP information and progress bars (Figure 5C and D) aim to motivate continuous gameplay.

To improve the notification system, we added support for email to deliver important notifications (eg, match begins or ends) and created new types of notifications, such as for when a 4-in-a-row is taken, a cell is taken, another team’s 4-in-a-row chance is blocked, the player’s team’s 4-in-a-row chance is blocked, a match is started, a match is ended, a player achieves a Lucky Cell or a Task Master, and a player achieves a new rank. The player can opt in or opt out of notifications (Figure 5E) and view the notification history (Figure 5F) that summarizes all activities in a match.

As the competition was disliked by the evaluation participants, we implemented a 1-team match mode where the players collaborate to meet the ending conditions as soon as possible. This mode serves as a basis for future tasks that are based on collaboration.

Finally, we implemented log-in through Facebook or Google accounts. Deeper social media integration (eg, contact lists and discussion forums) remains to be implemented. We anticipate that integrating social media accounts into the game will make it more convenient for players to play and create new possibilities for future feature expansion and interconnectivity with other systems.

### Children’s AST Ideas and Road Map for Future Development

In addition to developing new features based on the formative evaluation, we asked another group of pupils (13/33, 39% male and 20/33, 61% female) what features they would like to see in the AST game. We then mapped the gathered ideas to the eight core drives of gamification proposed by the Octalysis gamification framework [30]—meaning, empowerment, social influence, unpredictability, avoidance, scarcity, ownership, and accomplishment—and created a road map of future features for Tic-Tac-Training. The results are presented in Multimedia Appendix 2 [30].

## Discussion

### Principal Findings

We presented the conceptual and technical details of the Tic-Tac-Training game, which was designed to promote active transport at workplaces. The original game was based on competition between teams and offered little incentive for continuous gameplay over multiple matches. By analyzing the subjective perceptions gathered through a formative evaluation with schoolchildren in northern Sweden, we discovered that this approach is not well-suited for schoolchildren as they generally prefer collaboration to competition and have different opinions from those of adults regarding what a good game should be like. Thus, based on the formative evaluation results, we developed a new version of the game with new features that aim to make the game more interesting and appealing to schoolchildren, incentivize long-term engagement in gameplay, and increase collaboration. In addition, we collected ideas for the gamification of AST in general from another group of schoolchildren through focus group interviews and mapped the findings to the Octalysis gamification framework. On the basis of these ideas, we derived a road map for future Tic-Tac-Training features.

### Voluntary Engagement and Motivation

In the formative evaluation, we asked the participants for their opinions on the expected impact of the game on fellowship, physical activity, and the environment, which are the underlying themes of the game. The results were mostly negative as shown in Table 3. A possible explanation for this is that the participants played the game only once; a longitudinal study could reveal different results and detailed reasons. Another possible explanation is that the game failed to motivate the participants to play the game *minds-on*, and the participants considered it merely a task that the teacher gave them. This explanation is supported by a participant’s comment: “Our teacher made us play it” (Male, 11). An important lesson can be learned here: players may see the impact of an intervention more clearly if they are convinced to voluntarily engage in it in the long term. Exploring voluntary engagement in serious games in depth is a topic for another study. However, gamification has been shown to support a myriad of factors that can promote intrinsic motivation and engagement [28]. Moreover, motivational theories can help explain the presence or absence of essential motivational components in the game. For example, the components of the self-determination theory [55]—autonomy, competence, and relatedness—were not fully supported by the original version of Tic-Tac-Training that the schoolchildren played. The game supported limited autonomy by allowing players to make arbitrary moves on the game board. Support for autonomy could be increased by allowing players to customize their profiles and avatars, and create their matches. Moreover, adding XPs and levels can be seen as ways to promote competence, and collaboration features such as team play, 1-team matches, and chats support relatedness. A future longitudinal evaluation may show how well the new features of Tic-Tac-Training will support voluntary engagement and motivational theories such as the self-determination theory and whether there are any changes to the perceived impacts gathered in the formative evaluation.

### Social Media Connection: Opportunities and Threats

One of the new features added to Tic-Tac-Training was linking social media accounts such as Facebook and Google through the OAuth authorization protocol. This linkage provides a basis for further use of social media in the game through importing friend lists, integrating social media–based discussions, and even integrating the game onto a social media platform such as Facebook. Such social media integration was also suggested by children who gave ideas to gamify AST. The ability of the game to connect to a popular social media platform increases its potential for large-scale adoption. This introduces threats such as privacy and safety in web-based environments that one cannot ignore. Most social media accounts have age limits for children, but it is not uncommon for children under the age limit to become avid users of social media. For example, according to a report by Ofcom, 30% of children aged 5-7 years and 44% of children aged 8-11 years used social media in the United Kingdom in 2020 [56]. Using publicly available information makes it possible to build detailed profiles of people that can be used for criminal purposes [57]. Furthermore, social media can become a context for cyberbullying [58]. When integrating social media accounts into Tic-Tac-Training, the same precautions must be taken as with social media use in general. To this end, many social media privacy guides exist, such as the social media privacy guides offered by Internet Matters [59], a nonprofit organization dedicated to promoting children’s safety in web-based environments.

### Gamification Potential

The road map of the new Tic-Tac-Training features presented in Multimedia Appendix 2 forms the basis for the next development steps. The ideas mapped well to the core drives of gamification [30]; only one of the drives, unpredictability, was left without ideas from the children. Overall, the collected gamification ideas indicate that schoolchildren have the capacity to develop diverse ideas based on their knowledge of and experience with different games. This supports previous observations that children possess assets as co-designers of games, such as creativity, relationships, and emotional impacts [60]. Although it is unnecessary to support all core drives for a game to be successful, supporting many of them can help the game reach different player types within the same age group who have different preferences about playing games. There exist several models for player types that can be useful for understanding differences between players, such as Killers, Achievers, Explorers, and Socializers by Bartle [61]; the 4 temperaments by Keirsey [62]; the Demographic Game Design 2 model by Bateman et al [63]; and the Hexad model by Marczewski [32]. A future iteration of Tic-Tac-Training could use one or more of these models to identify each player’s type and adjust the gameplay accordingly. For example, the task types could be automatically customized to use the types of game mechanics that the player enjoys.

As the features proposed in the road map are derived from children’s ideas, we expect that they can help increase the game’s motivation, appeal, and engagement factors among Swedish schoolchildren. In particular, having an evolvable avatar in the game seems important to many schoolchildren. Having a personalizable avatar supports the autonomy component of the self-determination theory [55] as the player voluntarily controls their avatar and its evolution. The competence component is promoted when the player develops the avatar’s skills by completing tasks and collecting gameplay data (eg, via sensors). Autonomy and competence are identified as basic psychological needs through which intrinsic motivation can be nurtured [55]; thus, a future version of the game has increased potential for intrinsic motivation. However, this is mere speculation until the road map features are implemented and their effects are measured in a future study.

As demonstrated in Multimedia Appendix 2, the use of avatars was a way to establish a deeper meaning for the gameplay. Developing and caring for an avatar motivates continuous gameplay, an important factor in supporting behavior change over time. Another potential gamification element related to establishing a more profound meaning is the use of narratives. Although they were not present in the children’s ideas, narratives and storytelling are key motivators in many games [28]. Moreover, research has shown that narratives can help increase physical activity among children [64].

Another road map idea that the children frequently brought up was the inclusion of an in-game currency that can be used to purchase items and develop the avatar. This idea is familiar, with many free-to-play mobile games offering in-game purchases of items and skills with real or in-game currency. The ability to earn in-game currency connects to competence as it is a direct result of successfully completing tasks, whereas making purchases in the game world supports the player’s autonomy. If the purchases were shared with others (eg, by decorating the player’s avatar and sharing it with other players), relatedness in the self-determination theory would also be supported. The demand for relatedness was also evident in the ideas of creating and sharing content (eg, photographs) and interacting with other players through social media.

### Limitations

Despite a number of contributions, this study has several limitations. First, although 16 is deemed to be a sufficient number of participants for identifying problems in a user test [50], the results cannot draw deeper conclusions on the impacts of the game. Second, the sample comprised only Swedish pupils in sixth grade. Although the context of the study was Sweden, having a more diverse set of participants from different countries and age groups would make the results more generalizable. We hypothesize that the results can be applicable at least in other northern regions of the Nordic countries as they are in many ways similar to Luleå, but this remains to be tested. Third, the updated version of the game was not evaluated to measure the effects the new features had on schoolchildren’s perceptions of the game. The formative evaluation was conducted in February 2020, before the global COVID-19 outbreak. Because of the pandemic and restrictions, we did not have a chance to evaluate the new features in this study.

## Conclusions and Future Work

The study’s primary objectives were to investigate the game’s suitability for promoting AST in schoolchildren, improve the game according to the results, and prepare a road map for future development based on ideas gathered from schoolchildren. We achieved these objectives by contributing to implementing the Tic-Tac-Training game, its formative evaluation and consequential upgrade, and additional gamification ideas elicited from schoolchildren. The study results are generalizable to other locations similar to the study’s context: a midsized city with harsh winter conditions and a well-maintained pedestrian and bicycle road network.

The results have potential impacts on how gamified AST interventions can be developed and what kind of gamification elements schoolchildren in the Swedish context wish to see in such interventions. The study results can be of interest to game researchers, developers, and teachers who wish to apply gamification to AST or other school subjects. For example, following the road map of features, a future game developer targeting elementary school contexts might design the gameplay around collaboration, avatars, rewards, and achievements. Finally, the proposed architecture can be useful for developers who wish to create reusable, collaborative game platforms on cross-platform web technologies.

However, the results are not conclusive in terms of verifying whether the new version of Tic-Tac-Training sufficiently addresses the issues discovered in the formative evaluation. Therefore, in future work, we plan to conduct a longitudinal mixed methods evaluation with the current version of the game once the COVID-19 pandemic is over. This evaluation will occur in multiple schools in Sweden and possibly other countries, such as Finland and the Republic of Korea, and will involve children from grades 3 to 9. In addition, we will start implementing the road map features and developing more collaborative games for AST. Finally, based on a body of literature and our experiences, we seek to develop a model for designing collaborative gamification interventions for AST.

## Appendix

Multimedia Appendix 1 Formative evaluation questionnaire (translated).

Multimedia Appendix 2 Tic-Tac-Training development road map.

## Abbreviations

API: application programming interface
AST: active school transport
UI: user interface
XP: experience point
