# Supplementary figures and images for: A Reusable Multiplayer Game for Promoting Active School Transport: Development Study
Source: JMIR Serious Games. 2022 Mar 14;10(1):e31638. doi: 10.2196/31638 (PMC8961339; doi:10.2196/31638)

## Multimedia Appendix 1: Formative Evaluation Questionnaire (translated)


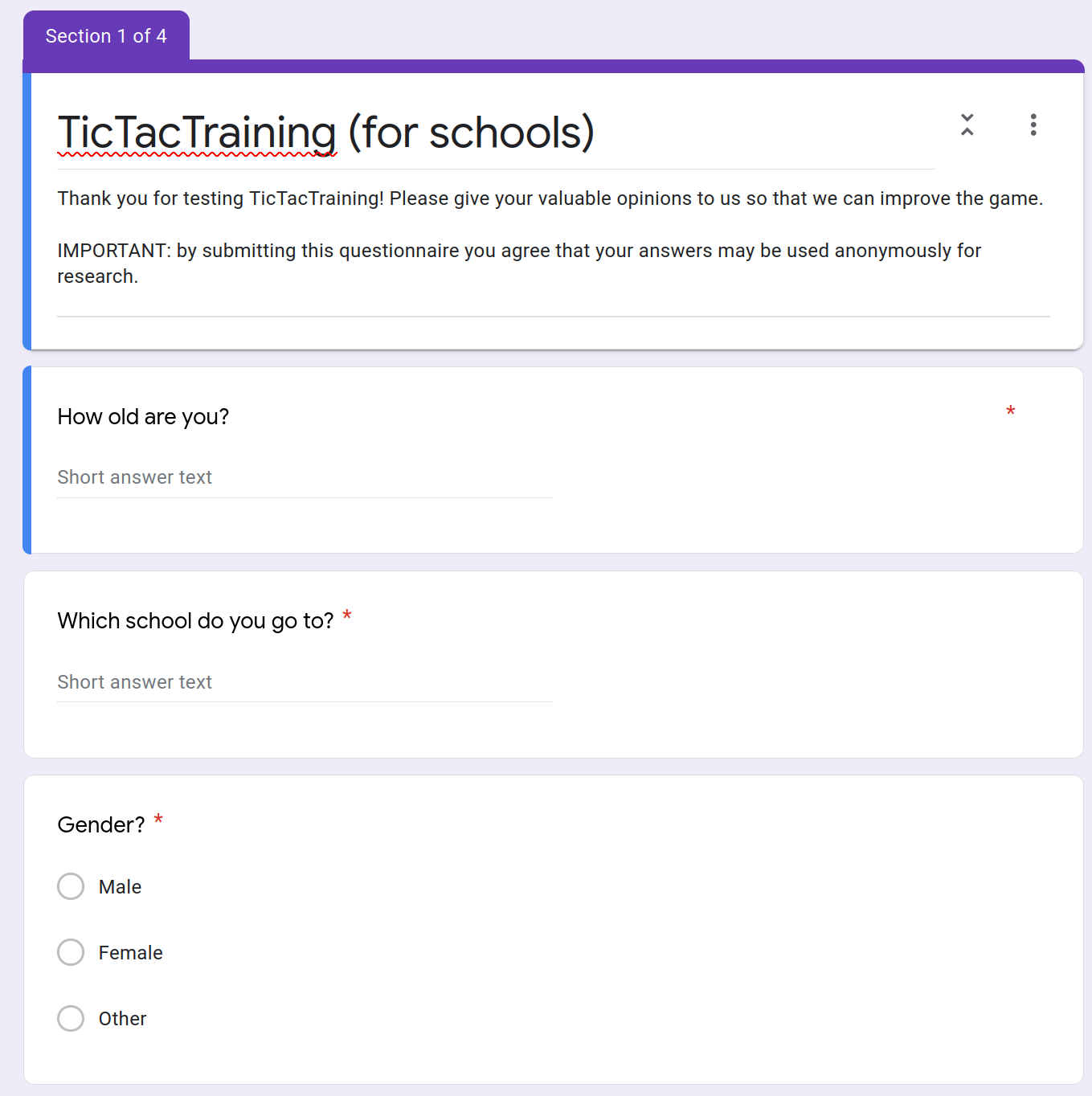


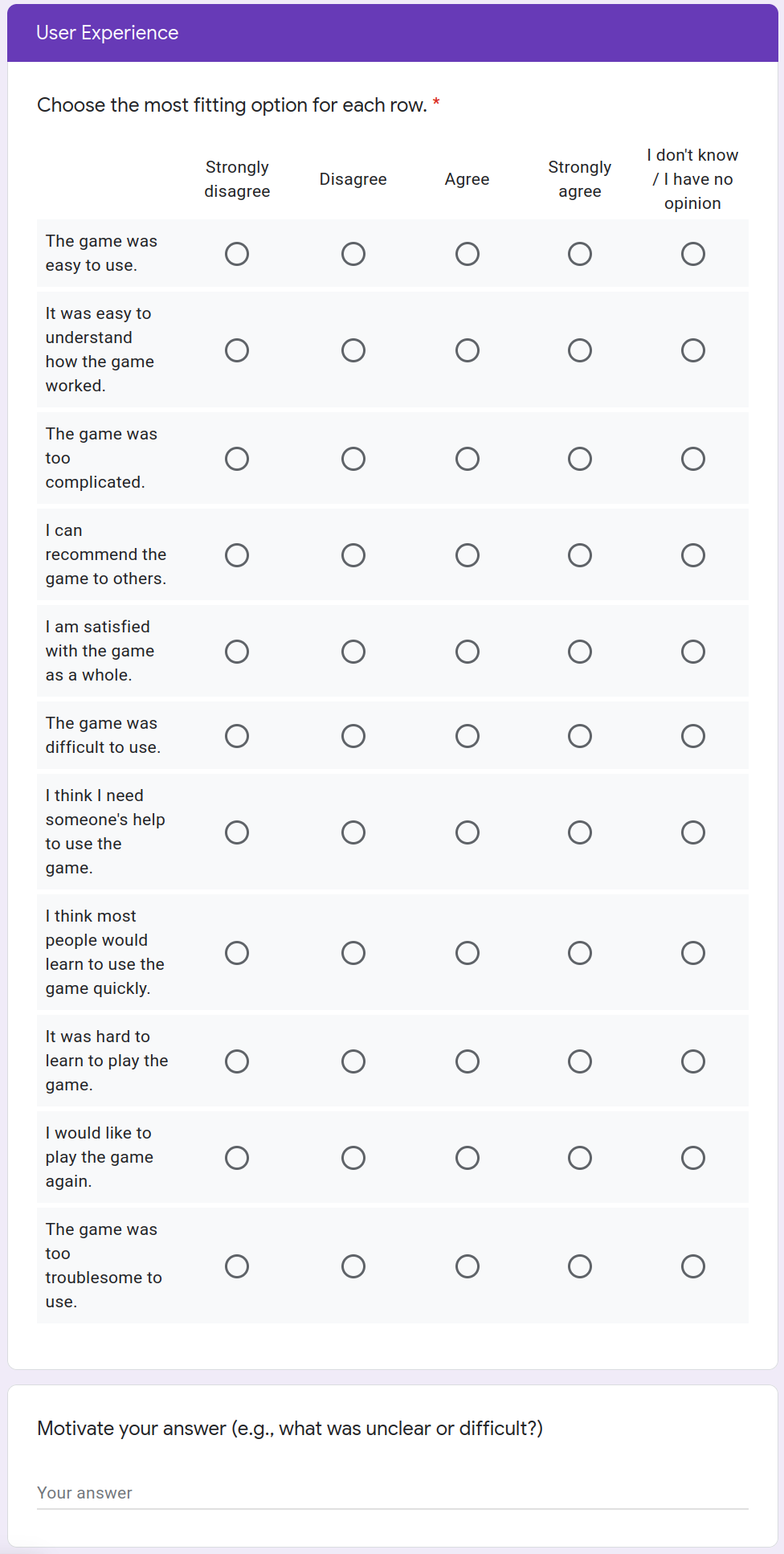

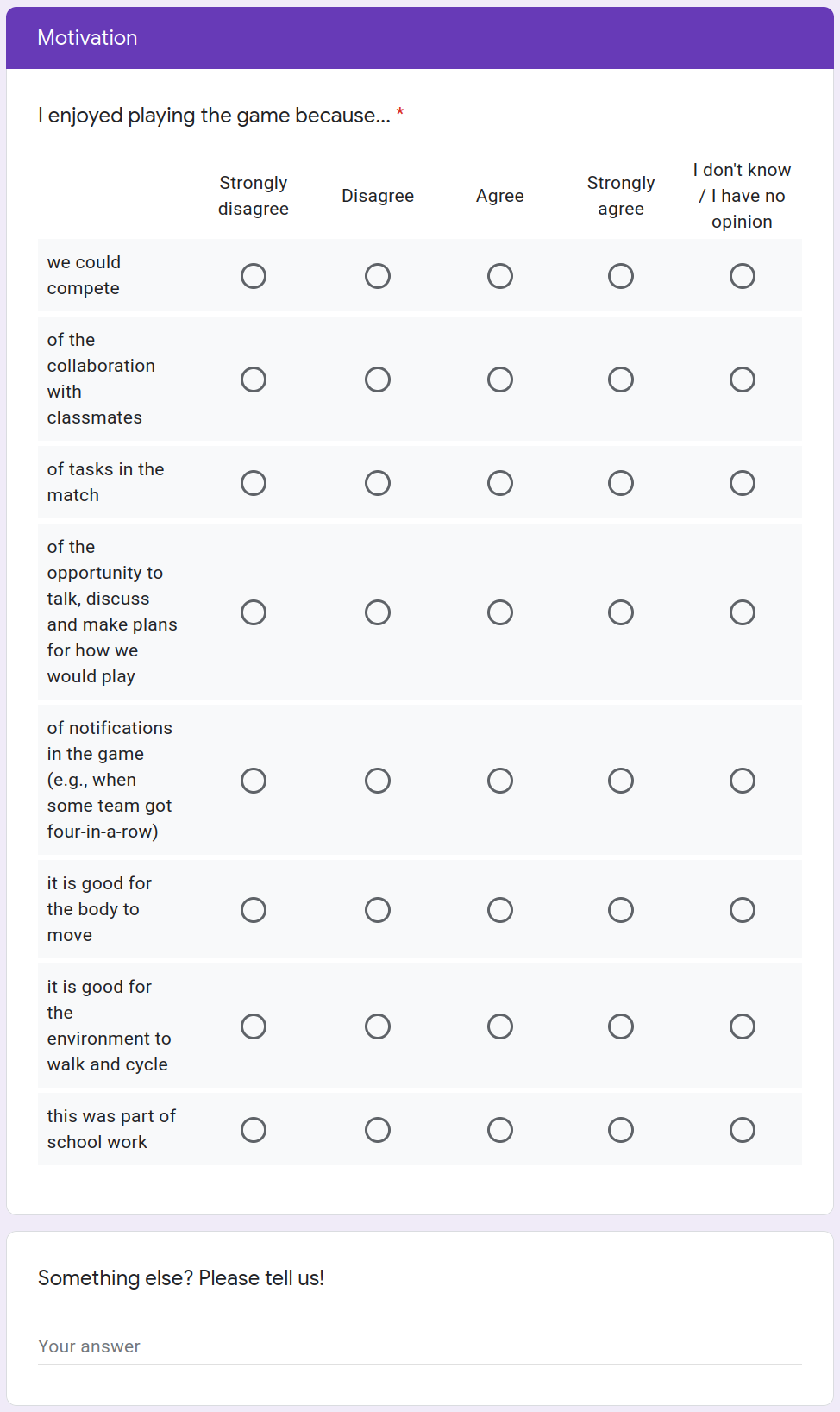

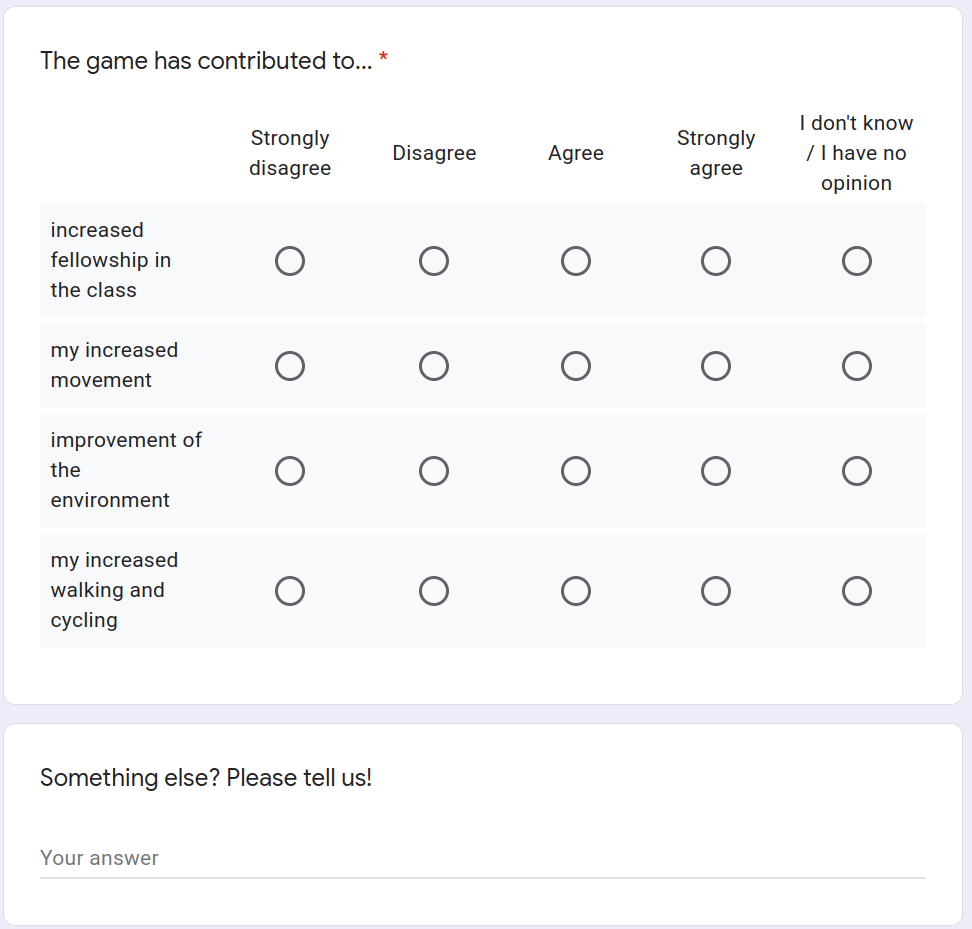


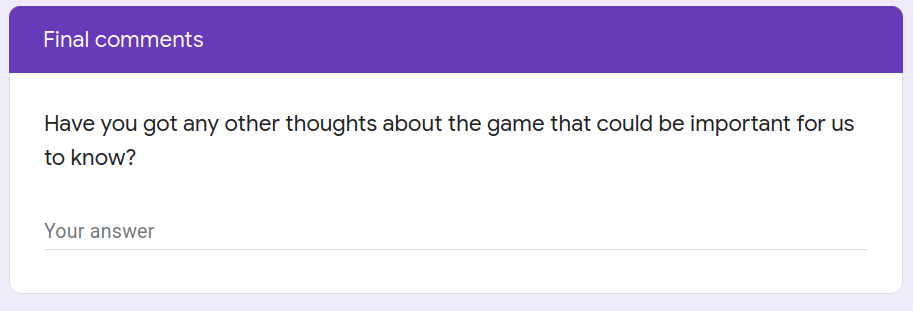

Supplement: Multimedia Appendix 1 [file games_v10i1e31638_app1.docx]
