# Supplementary material for: A Reusable Multiplayer Game for Promoting Active School Transport: Development Study
Source: JMIR Serious Games. 2022 Mar 14;10(1):e31638. doi: 10.2196/31638 (PMC8961339; doi:10.2196/31638)
Supplement: Multimedia Appendix 2 [file games_v10i1e31638_app2.docx]

## Multimedia Appendix 2: Tic-Tac-Training Development Road Map

**Table S1.** Tic-Tac-Training development road map based on schoolchildren’s ideas and the 8 core drives of gamification [30].

| Core drive and ideas from the children | | Road map features | |
| --- | --- | --- | --- |
| **Meaning** | | | |
|  | Having an avatar in the game; having an avatar in the game that also goes to school by bicycle or walking | The game could have a story where the protagonist is an avatar that the player *owns* and can identify with. There could be tasks in the game that connect to the game story. The meaning comes from this relationship between the player and the avatar and the ultimate goal that the avatar tries to reach (eg, save the environment). The player’s success in tasks could gradually grant the avatar different ranks of a hero, thus further strengthening this core drive. | |
|  | Having quests in the game such as going biking while collecting points and avoiding dangers; assignments in the neighborhood (eg, taking a picture of something, checking in at Konsum [supermarket], or running for 4 minutes) | | Development of more diverse tasks that are aware of the user’s context. For example, the game could have location-based tasks connected to the physical-world locations or tasks that measure the player’s physical activity and transform that into information (eg, points) that can be used in the game environment. |
| **Empowerment** | | | |
|  | Post pictures and receive attention; measuring physical activity | Empowerment can be nurtured by providing the player with methods to create and share content (eg, photographs, comments, and videos) and allowing them to collect performance data that can be visualized and used in the tasks. Moreover, based on the created content, collected data, and performed tasks, the game could have instant, multidimensional feedback (game-to-player, player-to-player, and teacher-to-player) that guides and adapts the game experience. | |
|  | Having an avatar in the game and caring for it, buying things (eg, clothes, food, and houses) with your earned coins; earning points and being able to upgrade your avatar (eg, buying a faster and cooler bicycle, winter tires, or a snowplow to attach in front of the bicycle) | When the player moves from one match to another, they can purchase, produce, and acquire digital artifacts (eg, photographs taken during a match and special equipment or skills earned) through which they are empowered to change the gameplay experience. The artifacts could then be used to decorate and equip the avatar. Some of the artifacts could be power-ups that give advantages when completing tasks. | |
| **Social influence** | | | |
|  | Socialize with others through social media (Facebook, Instagram, and Snapchat); create a Snapchat or Instagram account for the project and receive a lot of likes and followers; post pictures and receive attention; display pictures on screens at school (eg, in the lunchroom) | Although the game has connections to social media service provider accounts (Facebook and Google), the social media features have not yet been integrated into the game, nor does it have Snapchat or Instagram integration. The next version of the game could support social influence through various means (eg, improved chat by linking it to social media, friend list based on social media, and artifact or experience sharing). There could also be mentorship through which a more experienced player (or teacher or parent) could guide a novice. The mentor could also be a virtual character representing the game on social media and connecting to the players. The virtual mentor could also be integrated into the chat feature through a machine learning–based chatbot that provides context-sensitive responses and guides to players. | |
|  | If you walk or cycle together, you earn extra coins; the class plays together collecting coins | The game could have tasks that require collaboration to be successfully completed. The collaboration could be asynchronous (ie, each player can do the task on their own time within a given time interval) or synchronous (ie, the players do the task at the same time). If the game measured the players’ physical movement, the collected data could determine whether the users perform the task collaboratively. | |
|  | Being able to meet and interact with other avatars; competing against other schools, classes, or individuals | Although the competition was disliked by most of the participants, some schoolchildren found it appealing. The game could compete against other classes and schools, thereby promoting in-class fellowship. Moreover, the game could offer opportunities for the players to interact with other players through their avatars, perhaps by expanding the game world into the realm of social media. | |
| **Unpredictability** | | | |
|  | None recorded | The game content should not be predictable. Unpredictability can be achieved by making each gameplay experience unique by considering the player’s background, preferences, and context (eg, physical and environmental) in which the game is played and randomizing some aspects of the game tasks based on predefined task templates. For example, a task template requiring the user to walk 20 minutes could be contextualized into a task to walk to a nearby river, take a photograph of the sunset, and walk back, thus considering the player’s location and time of day. | |
| **Avoidance** | | | |
|  | If you go by car, you will lose coins; if you are out of coins, your clothes and things might be confiscated; having quests in the game such as going biking while collecting points and avoiding dangers | Avoiding losing something precious or avoiding entering into a dangerous situation can be powerful motivators. Therefore, the game could harness this core drive through making the collected or constructed artifacts precious (eg, powerful effect) but fragile (eg, expiration date that can be extended by active gameplay or inflicting damage through failures in tasks). | |
| **Scarcity** | | | |
|  | Special quests where you must act fast to be able to obtain the coins (eg, only the first 5 to do the assignment obtain the coins) | Related to avoidance, scarcity of certain game artifacts can make their discovery a rewarding experience. This core drive is already supported through Task Master and Lucky Cell rewards. More scarce items can be added to the game; for example, special artifacts for an avatar to wear or equip. | |
| **Ownership** | | | |
|  | Having an avatar in the game and caring for it, buying things (eg, clothes, food, and houses) with the coins you have earned | In learning and gaming, ownership of the experience is important. This can be achieved by allowing the player to own and customize their avatar as well as create, own, and use game artifacts (see *Empowerment*). | |
| **Accomplishment** | | | |
|  | Collect coins by walking or cycling; to be able to obtain cool things, you must earn a lot of coins (ie, walk or cycle a lot); assignments where you must do something several days in a row (eg, walk or cycle every day for 1 week); display pictures on screens at school (eg, in the lunchroom); measuring physical activity | Points, badges, the progress bar, and the leaderboard are some of the accomplishment elements found in many games for a good reason: they provide a feeling of achievement to the player. The game already includes a point system that is linked to the level system and ranks. To improve this, the game could have different kinds of points, such as experience points, exercise points, safety points, and environment points that are earned through different types of tasks. Apart from ranks, there could also be additional certificates or diplomas for achieving milestones and for performing certain actions (eg, collecting 10, 100, or 200 points). An avatar can be enhanced by allowing the player to demonstrate accomplishment by updating the attributes of the avatar. Finally, as accomplishments are often best shared, there should be a way to convey them to other players; for example, through social media. | |
